# Enzymes Inhibition and Antioxidant Potential of Medicinal Plants Growing in Oman

**DOI:** 10.1155/2022/7880387

**Published:** 2022-07-15

**Authors:** Najeeb Ur Rehman, Muddaser Shah, Saeed Ullah, Majid Khan, Ajmal Khan, Obaid Ullah, Javid Hussain, Ahmed Al-Harrasi

**Affiliations:** ^1^Natural and Medical Sciences Research Center, University of Nizwa, P.O. Box 33, Postal Code 616, Birkat Al Mauz, Nizwa, Oman; ^2^Department of Biological Sciences & Chemistry, College of Arts and Sciences, University of Nizwa, Oman

## Abstract

The recent study was designed to explore *Dodonaea viscosa*, *Juniperus excelsa*, *Helianthemum lippii*, and *Euryops pinifolius* using methanolic (MeOH) extract. Their subfractions were examined against urease, carbonic anhydrase II (CA-II), *α*-glucosidase enzymes, and free radicals scavenging significance based on local practices via standard methods. Significance potential against the urease enzyme was presented by ethyl acetate fraction (EtOAc) of *D. viscosa* with (*IC*_50_ = 125 ± 1.75 *μ*g/mL), whereas the *H. lippii* (*IC*_50_ = 146 ± 1.39 *μ*g/mL) in the EtOAc was found efficient to scavenge the free radicals. Besides, that appreciable capacity was observed by the *J. excelsa*, *D. viscosa*, *J. excelsa*, and *E. pinifolius* as compared to the standard acarbose (IC_50_ = 377.24 ± 1.14 *μ*g/mL). Maximum significance was noticed in methanolic (MeOH) extract of *J. excelsa* and presented carbonic anhydrase CA-II (IC_50_ = 5.1 ± 0.20 *μ*g/mL) inhibition as compared to the standard (acetazolamide). We are reporting, for the first time, the CA-II inhibition of all the selected medicinal plants and *α*-glucosidase, urease, and antioxidant activities of the *E. pinifolius*. Thus, further screening is needed to isolate the promising bioactive ingredients which act as an alternative remedy to scavenge the free radicals, antiulcer, and act as a potential source to develop new antidiabetic drugs for controlling postprandial blood sugar as well as carbonic anhydrase inhibitors.

## 1. Introduction

Phytomedicines are made up of medicinal plants and their chemical ingredients and have a key therapeutic role in various health-related complications, for instance, gastrointestinal infections, free radicals scavenging, and antidiabetic properties [1]. In this context, plant extracts are made up of a variety of chemical elements and are well-known for their wide range of clinical applications. They are derived from plants using both traditional and other modern approaches [2].

Urease enzymes play a leading role to catalyze the hydrolysis of urea, thus gaining substantial attention regarding human health and their life qualities [3–5]. It maintains optimum pH and treats the NH_3_ to balance its medium level due to which they own an incredible medical position [6, 7]. It is the main public health matter related to the bacterium *H. pylori*, which can endure in an acidic environment of the stomach having pH 2 range [6, 8]. The high prevalence of *H. pylori* in the human population indicates that such microbes have developed mechanisms for resistance against host defenses [8]. Marketed available urease drugs (phosphorodiamidate, hydroxamic acid derivatives, and imidazoles) are much more toxic with less efficacy rate and thus influenced by their limited clinical use [9, 10]. Thus, the quest for innovative urease inhibitors with enhanced stability and minimal toxicity is needed to improve the life quality of human beings and animals. Therefore, plant-based drugs are the alternative basis for having the ability to overcome the mentioned complications.

Diabetes mellitus (DM) is a common metabolic illness that has become a serious worldwide health issue. When DM is left untreated, it can harm the nerves, eyes, kidneys, and other organs. Increased urination, impaired vision, weariness, hunger, and thirst are among the symptoms of T2DM [11]. Controlling postprandial hyperglycemia by delaying carbohydrate digestion and absorption is one of the therapy options for T2DM. *α*-Glucosidase (EC 3.2.1.20) is an enzyme found on the small intestine's brush edge*. Inhibition of the α-glucosidase can limit the digestion of carbohydrates resulting in declined postprandial blood sugar levels* [12]. As a result, *α*-glucosidase inhibitors (AGIs) can be used as first-line therapy for T2DM [13–15].

Natural antioxidants can improve food quality including color, taste, flavor, and stability and also act as standardized nutrients (nutraceuticals) to end up the attack of free radicals in biological systems [16, 17], that might deliver additional health benefits to consumers [18, 19] and reduce the risk of disorders caused by free radicals [20]. Recently, considerable attention focused on the use of natural antioxidants to defend the human body against brain tissues and neurological disorders associated with free radical damage [20, 21]. This research is focused on searching for new sources of natural antioxidants and urease inhibitors that can be used directly or in combination with other official drugs as a lead compound for drug discovery.

Carbonic anhydrase (CA) is a metalloenzyme that contains zinc and is mainly used to catalyze CO_2_ hydration into bicarbonate and hydrogen ions [22]. The CA inhibitors control the enzymatic actions and prevent bicarbonate reabsorption which leads to numerous adverse effects such as potassium and bicarbonate retention in the human urine and decreased sodium absorption as a diuretic [23]. The intake of synthetic drugs for longer use to release this complication might be harmful. Therefore, searching for plant-based alternative remedies can be useful to cope with these disorders [24].

*Juniperus excelsa* M. Bieb (JE, Cupressaceae) is used mainly for lowering blood pressure [25], jaundice, bronchitis, tuberculosis, common cold [26], diabetes, stomachache, grazing, and wood harvesting [27]. Literature surveys documented its positive effects in treating colds, cough, dysmenorrheal, persuading menses, and expelling fetuses [28, 29]. *Dodonaea viscosa* Linn (DV, Sapindaceae) was reported to possess antiviral, anti-inflammatory, laxative, spasmolytic, antimicrobial, hypotensive agents [30], smooth muscle relaxant, anesthetic, throat infection, malaria, and antiulcerogenic [31, 32]. Traditionally, it is used to treat many illnesses like malaria, cold, aches, fever, toothaches, rheumatism, headaches, ulcers, diarrhea, dysmenorrhea, irregular menstruation, and constipation [33].

*Helianthemum lippii* (HL) belongs to the genus *Helianthemum*, which is a widely distributed and most taxonomically complex genus of the Family Cistaceae. Alsabri et al. [34] reported anti-inflammatory and analgesic activity of *H. lippii* against carrageenan-induced paw edema and hotplate-induced pain in rats. Previous studies showed that the plant is a rich source of polyphenols and flavonoids [35, 36] with antioxidant, antiulcer, and antimicrobial [37], as well as cytotoxic [38]. *Euryops pinifolius* A. Rich belongs to the genus *Euryops* (family: Asteraceae) [39], mostly cultivated in southern Africa, with a few species in other parts of Africa and on the Arabian Peninsula [28]. The local uses of *E. pinifolius* are least known, however, in some places of the Arabian Peninsula (Yemen, Oman, and Saudi Arabia), are used to wound healing [40].

To devise innovative plant-derived drugs, four important medicinal plants were collected from Al Jabal Al Akhdar (Northern Oman) and evaluated for antioxidant and enzyme inhibition activities. To the best knowledge, this is the first report on the enzyme inhibition study of these plants. In addition, we are also reporting the antioxidant activity of *H. lippii* and *E. pinifolius* for the first time.

## 2. Materials and Methods

### 2.1. Collection and Identification of the Medicinal Plants

Aerial parts of the plant species ,viz., *J. excelsa* (3.5 Kg), *H. lippii* (3.5 Kg), *E. pinifolius* (4.0 Kg), and *D. viscose* (5.5 Kg), were collected from Al Jabal Al Akhdar, Oman, identified by a plant taxonomist (Dr. Syed Abdullah Gillani) at the Department of Biological Sciences and Chemistry, University of Nizwa, Oman. After documentation, voucher specimens (HL-01/2012, EP-02/2012, DV-03/2012, and JE-04/2012) were kept at the herbarium of Natural and Medical Sciences Research Center, University of Nizwa, Oman, for further processing.

### 2.2. Extraction and Fractionation

**Figure 1:**
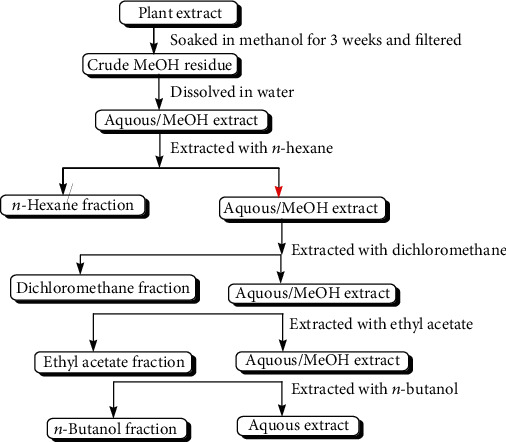
General fractionation scheme for the solvent-solvent extraction of medicinal plants.

The whole aerial parts of the *Dodonaea viscosa* were dried, chopped, and soaked in methanol at room temperature for 15 days three times as reported earlier by Shah et al. [16]. Evaporation of the MeOH in vacuo at 45°C yielded a crude methanol extract, which after suspension in water was successively fractionated into *n*-hexane, dichloromethane (CH_2_Cl_2_), ethyl acetate (EtOAc), butanol (n-BuOH), and aqueous (H_2_O) (Figure 1). The same procedure was used for the extraction and fractionation of other medicinal plants. The details of quantity of crude extracts and the different fractions of the selected plants are given in Table 1. The crude extracts and their fractions were subjected to biological screening to determine their potential effect.

### 2.3. Antioxidant Activity

The antioxidant bioassay of the crude extracts and subfractions of the selected plants was evaluated using *α*, *α*-diphenyl-*β*-picrylhydrazyl (DPPH) assay [41]. About 100 *μ*L of methanol was mixed with 150 *μ*L of DPPH solution as a negative control. For the sample, about 150 *μ*L of DPPH was added with 100 *μ*L of three concentrations of extract (100, 500, and 1000 *μ*g/mL). The absorbance was taken at 517 nm by spectrophotometer. All the results were compared to a control containing 50 *μ*L of methanol. The positive control used was ascorbic acid (Table 1). Each test was repeated three times, and % inhibition was calculated as:

(1) $$\begin{array}{c} \text{Percent }\left(\%\right)\text{ inhibition of DPPH activity}:A_{c}–A_{s}/A_{c}\times 100,\\ A_{c}=\text{Absorbance of control }\left(\text{ascorbic acid}\right);A_{s}=\text{Absorbance of sample}. \end{array}$$

### 2.4. Urease Enzyme Inhibition

Urease enzyme inhibition assay was performed using available literature [42, 43]. About 25 *μ*L solution of Jack bean urease was mixed with 50 *μ*L urea dissolved in phosphate buffer (pH 8.20) with 20 *μ*L of three concentrations (100, 500, and 1000 *μ*g/mL) of different extract fractions and then incubated at 37°C for 15 min. Then, 50 *μ*L of solution B (phenol reagent (1% w/w phenol) own expect 3 mg in 30 mL + 0.005% w/v sodium nitroprusside) and 70 *μ*L of solution A (alkali reagent (0.5% w/v NaOH +1% active chloride NaOCl)) were added and then incubated again for 15 min. Thiourea was used as a positive (standard) control, while methanol was used as a negative control (Table 2). The absorbance was recorded at 630 nm with a total volume of 215 *μ*L.

(2) $$\begin{array}{c} \text{O}{\text{D}}_{\text{test compound}}=\text{Optical Density of control }\left(\text{thiourea}\right);\text{O}{\text{D}}_{\text{control}}=\text{Optical Density of the sample}. \end{array}$$

### 2.5. *α*-Glucosidase Assay

All the twenty-four samples of crude extract and subfractions were evaluated in vitro against *α*-glucosidase enzyme (E.C.3.2.1.20) as described earlier by Shah et al. [11], by using (50 mM) phosphate buffer of pH (6.8). The enzyme was properly dissolved in the phosphate buffer; 1 U/2 mL, 20 *μ*L/well of the enzyme, and 135 *μ*L/well phosphate buffer was used as reaction buffer, 20 *μ*L/well of the tested samples were solubilized in DMSO (0.5 *μ*g/mL), in 96-wells plates incubated for 15 min at 37 °C. After the incubation period, the substrate para nitro phenyl-D-glucopyranoside was added at a concentration of 0.7 mM, and the change in absorbance was measured at 400 nm for 30 minutes. The positive control used was acarbose, and DMSO was used as negative control.

(3) $$\begin{array}{c} \text{O}{\text{D}}_{\text{test compound}}=\text{Optical Density of control }\left(\text{acarbose}\right);\text{O}{\text{D}}_{\text{control}}=\text{Optical Density of the sample}. \end{array}$$

### 2.6. Carbonic Anhydrase II Inhibition Assay

The total reaction mixture comprised 20 *μ*L (0.5 mmol/well) of test compounds (10% DMSO in total), and then add HEPES–Tris buffer 140 *μ*L (20 mmol, pH =7.4), 20 *μ*L of purified bovine erythrocyte CA-II (1 mg/mL, 0.1 units/well) prepared in buffer, and finally substrate 4-nitrophenyl acetate (4-NPA, 0.7 mmol) 20 *μ*L to attain final volume 200 *μ*L/well [44, 45]. An enzyme (EC 4.2.1.1, Sigma-Aldrich, St. Louis, MO, USA) along with the tested compounds was incubated for 15 min in a 96-well plate. Then, the reaction was started with the addition of 20 *μ*L of the substrate (4-nitrophenyl acetate) and continuously monitored the rate (*velocities*) of product formation for 30 min with the intervals of 1 min, at 25°C by using a microplate reader (Bio-Rad, Molecular Devices, CA, USA). Acetazolamide and DMSO were used as positive and negative controls, respectively.

(4) $$\begin{array}{c} \text{O}{\text{D}}_{\text{test compound}}=\text{Optical Density of control }\left(\text{acetazolamide}\right);\text{O}{\text{D}}_{\text{control}}=\text{Optical Density of the sample}. \end{array}$$

### 2.7. Statistical Analysis

The SoftMax Pro package and Excel were utilized.

The given formula below was used to calculate percent inhibition.

(5) $$\begin{array}{c} \%\text{Inhibition}=100-\left(\frac{\text{O}.{\text{D}}_{\text{test compound}}}{\text{O}.{\text{D}}_{\text{control}}}\right)\times 100. \end{array}$$

EZ-FIT (Perrella Scientific, Inc., USA) was used for IC_50_ calculations of all tested samples. To overcome on the expected errors, all experiments were performed in triplicate, and variations in the results are reported in standard error of mean values (SEM).

(6) $$\begin{array}{c} \text{SE}=\frac{\sigma }{\sqrt{n}}\#. \end{array}$$

## 3. Result and Discussion

Herbs have been used as a source of medicine since the dawn of human civilization, and they continue to play an important role in clinical use and quality control for a variety of health issues [46].

### 3.1. Antioxidant Capability

The antioxidant significance of the selected plants is determined to evaluate the free radical scavenger capacities of the selected plant species utilizing ascorbic acid as a standard inhibitor as presented in Table 2. In recent years, increasing attention has been paid to antioxidant compounds (flavones, anthocyanin, flavonoids, catechin, isoflavones, and other phenolics) derived from plants due to their valuable role in reducing various disorders such as immune system, brain dysfunction, heart disease, decline, cardiovascular disease, aging, and cancer [47]. The free radicals produced due to human metabolism affect the cellular membrane to overcome these complications [47]. The investigation reveals that among the screened four plant species, *H. lippii* fractions offered a significant ability to scavenge the free radicals and act as an antioxidant agent. The EtOAc fraction of *H. lippii* exhibited the highest potential to act as an antioxidant agent with IC_50_ of 146 ± 1.39 *μ*g/mL followed by the MeOH (IC_50_ = 368 ± 2.18 *μ*g/mL) and aqueous extract (460 ± 1.21 *μ*g/mL), respectively. This significance is attributed due to the presence of an affluent basis of polyphenolic constituents as documented by Benabdelaziz et al. [48]. Alali et al. [49] investigated *H. lippii* from Jordan and reported methanol (IC_50_ = 176.1 *μ*mol TE g^−1^dry weight) and aqueous (IC_50_ = 176.1 and 274.2 *μ*mol TE g^−1^dry weight) extracts in comparison to the standard via ABTS assay. However, in the current study, the geographical location, collection, habitat, harvesting season, screening approach, and standard used are different from earlier studies. Therefore, our finding reveals that the *H. lippii* has a significant ability to neutralize the free radicals. Belyagoubi et al. [36] collected *H. lippii* from Algeria as a plant habitat influenced the quality and quantity of bioactive compounds responsible for promising pharmacological potentials [50]. However, moderate capability was observed in the *n*-hexane and CH_2_Cl_2_ fractions (Table 2). In the case of *E. pinifolius*, the EtOAc fraction displayed significance inhibition (IC_50_ = 378 ± 1.56 *μ*g/mL) followed by the n-BuOH fraction (IC_50_ = 904 ± 2.64 *μ*g/mL). Moreover, the EtOAc fraction of *D. viscosa* also produced promising findings with (IC_50_ = 386 ± 1.65 *μ*g/mL) ensued by the n-BuOH (IC_50_ = 467 ± 1.84 *μ*g/mL), while normal activity was examined by the MeOH extract (Table 2). The current findings consented to the data stated for some Yemeni traditional medicinal plants by Mothana et al. [51] that *D. viscosa* was one of the most active plants that showed promising antioxidant activity. In addition to that, the current outcome also supports the results reported by Singh et al. [52] for *Rhus aucheri* as the understudy plant was collected from Oman. Recently, Muhammad et al. [53] isolated some flavonoids from the EtOAc fraction of *D. viscosa* showed higher antioxidant activity further stringent our findings. It was also observed that the free radicals scavenging significance of some medicinal plants from Iran was dissimilar from our recorded data as described by Boroomand et al. [54] due to the variation of their habitat, climatic, topographic, and edaphic factors influenced the content and quality of the metabolites accountable to act as an antioxidant agent. The data obtained from these *in vitro* models demonstrated the strong antioxidant potential of EtOAc and n-BuOH fractions of the selected medicinal plants, which might be a concern with its high medicinal and pharmaceutical use as a functional food in the treatment of different diseases.

### 3.2. Antiulcer Potential

The urease enzyme inhibitory activity of crude extracts/fractions of the plants was determined using a concentration of 1.0 mg/mL. Ethyl acetate fraction of *D. viscosa* exhibited significantly promising urease inhibition (IC_50_ = 125 ± 1.75 *μ*g/mL), followed by *n*-hexane (IC_50_ = _1_42 ± 2.00 *μ*/mL) and *n*-BuOH (IC_50_ = 410 ± 2.50 *μ*g/mL) fractions. The data of crude extract and fractions of *J. excelsa* revealed that only the EtOAc fraction exhibited significant inhibition (IC_50_ = _1_73 ± 2.50 *μ*g/mL) as compared to other fractions. In the case of *H. lippii*, the EtOAc fraction showed significantly strong inhibition (IC_50_ = 257 ± 1.25 *μ*g/mL), followed by n-BuOH (IC_50_ = 435 ± 2.75 *μ*g/mL), while MeOH and aqueous fractions of the same plant did not show activity (Table 3). The EtOAc fraction of E. pinifolius attributed promising inhibition (IC50 = 390 ± 2.50 *μ*g/mL), followed by the n-BuOH (IC50 = 430 ± 2.25 *μ*g/mL), while other fractions did not show urease inhibition (Table 3).

These findings provide crucial information about the biologically active constituents present in medicinal plants truly responsible for the inhibition of the urease enzyme. Thus, our finding matched with the data reported by Rauf et al. [55] for *Diospyros lotus* roots and in favor of the outcomes presented by Maherina et al. [56] as the use of the same approach to determine the urease significance in the medicinal plants. Moreover, our current findings do not agree with the data reported by Tahseen et al. [57] due to their variability in their habitat. In the future, bioassay-guided isolation of these secondary metabolites might be exciting and interesting to know the chemical constituents responsible for inhibition and to understand their basic mechanism against these enzymes.

### 3.3. Antidiabetic Significance

Crude extract and subfractions of the four plant species (*D. viscosa*, *J. excelsa*, *H. lippii*, and *E. pinifolius*) were tested to analyze their antidiabetic potential by targeting the key carbohydrate digestive enzyme *α*-glucosidase. Furthermore, the aqueous and n-hexane fractions of *J. excelsa* showed below 50% inhibitory activity and were found to be inactive. While other samples displayed several folds of potent inhibitory potential in the range of 1.30-20.75 *μ*g/mL, compared with acarbose (IC_50_ = 377.24 ± 1.14 *μ*g/mL). Moreover, the n-BuOH and n-hexane fractions of *D. viscosa* exhibited significant inhibitory activity with IC_50_ (1.30 ± 0.05 and 2.04 ± 0.06 *μ*g/mL), respectively. Thus, our data is supported by the literature explained by Assefa et al. [58] and VVM et al. [59]. It might be due to the presence of active chemical ingredients having the ability to cure diabetes. On the other hand, the EtOAc, DCM, aqueous, MeOH, n-hexane, and n-BuOH fractions of *J. excelsa* exhibited potent *α*-glucosidase inhibitory potential with IC_50_ (1.31 ± 0.02, 3.65 ± 0.12, 2.48 ± 0.13, 3.11 ± 0.14, 2.78 ± 0.11, and 2.05 ± 0.08 *μ*g/mL), respectively. Thus, our current screening consented to the findings of Bhatia et al. [60], which depicted a little variation in outcomes reported by Sancheti et al. [61] and Gok et al. [62]. Due to differences in the chemical ingredients influenced by environmental factors and the solvents and methods used in our studies. in addition to that, the *E. pinifolius* offered variations in the anti-*α*-glucosidase potential. For instance, the MeOH extract was found to be the most potent and displayed IC_50_ = 2.86 ± 0.03 *μ*g/mL. A slight decrease in the *α*-glucosidase inhibitory activity was observed in the other fraction samples, such as EtOAc having IC_50_ = 7.85 ± 0.16 *μ*g/mL. Likewise, a slightly further decline in the antidiabetic capability was observed, in the DCM and n-BuOH fractions depicted (IC_50_ = 16.72 ± 0.15 *μ*g/mL and 22.12 ± 0.15 *μ*g/mL), respectively. So, these outcomes also reflect that our data agrees with the findings of Khatib et al. [63].

Furthermore, our investigation exhibited a little variation as compared to the data stated by Ibrahim et al. [64] as mentioned previously that the habitat variation can also be led to variability among the chemical ingredients among the different and same plant species. Interestingly all the fractions of *H. lippii* displayed several fold potent inhibitory activities with almost the same potency comprises of EtOAc, DCM, aqueous, n-hexane, and BuOH with IC_50_ of 5.12 ± 0.18, 5.73 ± 0.21, 5.47 ± 0.13, 5.73 ± 0.21, and 6.45 ± 0.11 *μ*g/mL, respectively, as compared to MeOH extract (IC_50_ = 10.48 ± 0.26 *μ*g/mL). Our current study consented to the literature described by Zarei et al. [65]. However, our current result does not agree with the findings of Rungprom et al. [66]. The similarity of the antidiabetic significance presented by the medicinal plants might be due to the presence of the phenols and flavonoids. As we know in the current era plant extractions are becoming increasingly popular in medicinal therapies, and they are an alternative and valuable herbal medicinal medicine because of their broad usage and lower adverse effects, the current results insight into the crucial therapeutic importance of the *D. viscosa*, *J. excelsa*, *H. lippii,* and *E. pinifolius* in their crude and subfractions. Hence, these promising findings might be used as a therapeutic approach for the management of type 2 diabetes (*T2DM*) displayed in Table 3.

### 3.4. Carbonic Anhydrase II Significance

The selected plants are profiled for their carbonic anhydrase activity as shown in Table 3. Among the subfractions, EtOAc fraction of the *D. viscosa* presented significanse activity with IC50 of 27.5 ± 3.12 ± µg/mL trail by the MeOH extract IC50 = 50.4 ± 2.03 µg/mL, while other subfractions were found inactive. The *D. viscosa* contains phenols and polyphenols having the capability to act as carbonic anhydrase inhibitors; thus, our findings agree with the data stated by Karioti et al. [67]. The current finding presented that our data do not match with the data recorded by Rudenko et al. [68] because environmental stress influences the quality and quantity of bioactive ingredients responsible for numerous biological activities. Furthermore, the MeOH extract of *J. excelsa* followed by the EtOAc fraction of *J. excelsa* displayed significant potential an IC_50_ = 5.1 ± 0.20 and IC_50_ = 38.4 ± 2.52 *μ*g/mL significance in comparison to other tested fractions. The n-BuOH fraction of *E. pinifolius* presented appreciable significance having IC_50_ = 41.5 ± 0.82 *μ*g/mL, followed by the DCM fraction with IC_50_ = 45.4 ± 2.08, and the EtOAc fraction exhibited an IC_50_ = 47.0 ± 3.99 *μ*g/mL potential, whereas the MeOH and n-hexane extract were found inactive for carbonic anhydrase activity. The *H. lippii* fractions also displayed appreciable potential except for the DCM and MeOH extracts, while aqueous extract was most potent presented IC_50_ = 9.9 ± 0.35 *μ*g/mL, proceeded by the n-hexane with IC_50_ = 18.8 ± 3.13 *μ*g/mL and IC_50_ = 35.6 ± 1.32 *μ*g/mL in comparison to the standard acetazolamide having an IC_50_ = 4.04 ± 1.63 *μ*g/mL. The current results also match up with the outcomes of Aydin et al. [69] for *Satureja cuneifolia* and dodoneine by Carreyre et al. [70] which was found effective for the carbonic anhydrase activity. However, the study reported for the bioactive ingredient dodoneine by Carreyre et al. [70] was significant as compared to our current findings because might be bioactive compounds are responsible compounds as compared to our selected plants and tested fractions

## 4. Conclusion

In conclusion, the selected medicinal plants (D. viscosa, J. excelsa, H. lippii, and E. pinifolius) possess significance anti-ulcer, antioxidant, antidiabetic and carbonic anhydrase-II inhibition and can be considered as essential source of bioactive ingredients. Additionally, up to now, no such scientific data were reported for the enzyme inhibition potential, whereas the two plant species were reported for the first time in a recent study. Therefore, it could be contended that the medicinal plants have significant potential to serve as an antioxidant and own enzyme inhibitory attributes. However, further investigations are considered necessary for the isolation and identification of the chemical ingredients accountable for the antioxidant and enzymatic significance of the selected plants.

## Figures and Tables

**Table 1:** Crude extracts and fractions of the selected medicinal plants.

Plant's name	Crude (g)	Fractions (g)				
	MeOH	*n*-hexane	CH_2_Cl_2_	EtOAc	n-BuOH	H_2_O
*D. viscosa*	135	16	45	28	14	28
*J. excelsa*	115	11	23	20	24	35
*H. lippii*	95	12	18	25	12	28
*E. pinifolius*	90	13	19	21	18	17

**Table 2:** Antioxidant activity (%) of four Omani medicinal plants.

Plant Species	Fractions	Antioxidant activity IC_50_ ± S.E.M (*μ*g/mL)
*J. excelsa*	n-Hexane	Nd
	DCM	Nd
	EtOAc	402 ± 2.15
	n-BuOH	428 ± 1.54
	MeOH	Nd
	Aqueous	Nd
*E. pinifolius*	n-Hexane	Nd
	DCM	Nd
	EtOAc	378 ± 1.56
	n-BuOH	904 ± 2.68
	MeOH	Nd
	Aqueous	Nd
*H. lippii*	n-Hexane	788 ± 2.68
	DCM	658 ± 1.54
	EtOAc	146 ± 1.39
	n-BuOH	561 ± 1.34
	MeOH	368 ± 2.18
	Aqueous	460 ± 1.21
*D. viscosa*	n-Hexane	Nd
	DCM	Nd
	EtOAc	386 ± 1.65
	n-BuOH	467 ± 1.84
	MeOH	914.±2.61
	Aqueous	Nd
Ascorbic acid		_6.25 ± 0.56_

Ascorbic acid∗= *μ*M; DCM: dichloromethane; EtOAc: ethyl acetate; BuOH: n-butanol; MeOH: methanol; SEM: standard error mean; Nd: not determined (Conc. = 1 mg/mL).

**Table 3:** *α*-Glucosidase CA-II and urease activities of the selected medicinal plants.

Sample code	Fractions	*α*-Glucosidase IC_50_ ± SEM (*μ*g/mL)	Urease IC_50_ ± SEM (*μ*g/mL)	CA-II IC_50_ ± SEM (*μ*g/mL)
*D. viscosa*	EtOAc	5.99 ± 0.20	125 ± 1.75	27.5 ± 3.12
	DCM	10.86 ± 0.17	416 ± 1.50	NA
	Aqueous	5.34 ± 0.14	NA	NA
	MeOH	3.18 ± 0.10	NA	50.4 ± 2.03
	n-Hexane	2.04 ± 0.06	142 ± 2.00	NA
	n-BuOH	1.30 ± 0.05	410 ± 2.50	NA
*J. excelsa*	EtOAc	1.31 ± 0.02	173 ± 2.50	38.4 ± 2.52
	DCM	3.65 ± 0.12	NA	46.3 ± 1.95
	Aqueous	2.48 ± 0.13	NA	51.3 ± 1.35
	MeOH	3.11 ± 0.14	NA	5.1 ± 0.20
	n-Hexane	2.78 ± 0.11	NA	NA
	n-BuOH	2.05 ± 0.08	NA	66.8 ± 3.19
*E. pinifolius*	EtOAc	7.85 ± 0.16	390 ± 2.50	47.0 ± 3.99
	DCM	16.72 ± 0.15	NA	45.4 ± 2.08
	Aqueous	N/A	NA	98.2 ± 4.84
	MeOH	2.86 ± 0.03	NA	NA
	n-Hexane	N/A	NA	NA
	n-BuOH	22.12 ± 0.15	430 ± 2.25	41.5 ± 0.82
*H. lippii*	EtOAc	5.12 ± 0.18	257 ± 1.25	35.6 ± 1.32
	DCM	5.73 ± 0.21	NA	NA
	Aqueous	5.47 ± 0.13	NA	9.9 ± 0.35
	MeOH	10.48 ± 0.26	NA	NA
	n-Hexane	5.73 ± 0.21	NA	18.8 ± 3.13
	n-BuOH	6.45 ± 0.11	435 ± 2.75	59.4 ± 2.33
Acarbose		608.21 ± 1.74		
Thiourea			1.58 ± 0.95	
Acetazolamide				4.04 ± 1.63

DCM: dichloromethane; EtOAc: ethyl acetate; BuOH: butanol; MeOH: methanol; N/A (nonactive); concentration =0.5 mg/mL; SEM: standard error mean; ND: not determined.

## Data Availability

All datasets on which the conclusion of the manuscript relies are presented in the paper.
